# Physicochemical and Functional Characteristics of RD43 Rice Flour and Its Food Application

**DOI:** 10.3390/foods9121912

**Published:** 2020-12-21

**Authors:** Phim on Suklaew, Charoonsri Chusak, Sirichai Adisakwattana

**Affiliations:** Phytochemical and Functional Food Research Unit for Clinical Nutrition, Department of Nutrition and Dietetics, Faculty of Allied Health Sciences, Chulalongkorn University, Bangkok 10330, Thailand; Phim.on.b@gmail.com (P.o.S.); Charoonsri.c@gmail.com (C.C.)

**Keywords:** RD43 rice, physicochemical properties, starch digestibility, bile acid binding, cholesterol micellization, muffin

## Abstract

The increased use of a new rice cultivar is the result of increasing consumer demands for healthier choices. In this study, physicochemical, thermal, pasting, and functional properties of flour from RD43 rice, a new rice variety, and its food application were investigated. RD43 rice flour demonstrated an irregular and polyhedral shape with a volume mean diameter of 103 ± 0.15 µm. In addition, the amylose content of RD43 rice and Hom Mali rice flour was 19.04% and 16.38%, respectively. The X-ray diffraction (XRD) and Fourier Transforms Infrared (FTIR) confirmed the presence of a V-type crystalline structure and less crystallinity in RD43 rice flour, which resulted in a significant reduction of the water absorption index (WAI), swelling power (SP), water solubility index (WSI), gelatinization temperature, and pasting properties. Comparing with Hom Mali rice flour, RD43 rice flour had greater ability to disrupt cholesterol micellization and bind bile acid. Furthermore, it had lower starch digestibility, with a lower percentage of rapidly digestible starch (RDS) and higher percentage of undigestible starch than Hom Mali rice flour. Moreover, steamed muffins based on RD43 rice flour had lower starch digestibility than Hom Mali steamed muffins. The sensory analysis showed no significant differences between Hom Mali and RD43 steamed muffins. The findings suggest that RD43 rice flour could be an alternative ingredient for lowering the glycemic index of food products.

## 1. Introduction

Rice (*Oryza sativa*, L.), one of the most important crops in the world, is a principal staple food in many regions of the world’s human population, especially in Asia [1]. Today, there are more than 4000 identified rice varieties, which differ in their physicochemical and functional properties [2]. Apart from consumption as cooked granular form, rice flour is popularly used as a raw material for novel foods, because of its bland taste, high digestibility, hypoallergenic properties, and low price [3]. It has been shown that diversity in rice may include certain difference in granular size, amylose, lipid-complexed amylose, gelatinization, and starch composition, affecting the quality of rice flour and its products [4,5,6]. Studies on the physicochemical and functional properties of rice flour are necessary to indicate market value, consumer preference, and food utilization [4,7,8].

Hom Mali rice or jasmine rice, the most popular variety of any rice globally, has low apparent amylose content, which produces soft and sticky texture after cooking [9]. In particular, Hom Mali rice flour has been used in the formulation of flour-based products, such as bread and crackers [4,10]. Although Hom Mali rice provides good texture and mild floral aroma, it is considered a food with a high glycemic index (GI) [4]. Consumption of white rice contributes to a relatively large glycemic response associated with exacerbating impaired glucose tolerance. A meta-analysis revealed that over-consumption of white rice, categorized as a high-GI food, has been strongly associated with the development of non-communicable diseases, such as type 2 diabetes [11]. Therefore, several efforts have been made to develop a new rice variety with the improvement of physiochemical and functional properties, particularly to reduce the glycemic index. For example, riceberry rice, a cross-bred strain between Hom Nin rice (known as a high-antioxidant rice) and Hom Mali 105 rice, has demonstrated a lower starch digestibility and GI than Hom Mali rice because of the presence of anthocyanins and undigestible starch [4]. Recently, a new rice variety, RD43 rice (SPR99007-22-1-2-2-1 registered by the Rice Department, Thailand, 5 October 2010) was developed by crossbreeding between Khao Jow Hawm Suphan Buri and Supan Buri1. The RD43 rice is non-GMO and approved by the Rice Department of Thailand [12]. Most consumption of RD43 rice is as cooked rice served simultaneously with vegetables, poultry, and other dishes. However, there exists no available novel foods made from RD43 flour, due to limitations in its physiochemical and functional properties. Therefore, the objective of this research was to investigate physicochemical, pasting, thermal, functional properties, and in vitro starch digestibility of RD43 rice flour compared to Hom Mali rice flour. The application in steamed muffins made from RD43 rice flour was also evaluated.

## 2. Materials and Methods

### 2.1. Materials

RD43 rice (*Oryza sativa* L.) and Hom Mali 105 rice (*Oryza sativa* L.) were obtained from the Rice Department, Bangkok, Thailand. Pepsin from porcine gastric mucosa powder (250 U/mg), α-amylase Type VI-B from porcine pancreas (15.8 U/mg), pancreatin from porcine pancreas (4×U.S. Pharmacopeia (USP) specifications), pure amylose from potatoes, phosphatidylcholine, oleic acid, taurocholic acid, glycodeoxycholic acid, taurodeoxycholic acid, taurocholic acid, and porcine cholesterol esterase (35 U/mg) were purchased from Sigma-Aldrich, Inc. (St. Louis, MO, United States). Amyloglucosidase (3,260 U/mL) was purchased from Megazyme International Ireland Ltd. (Bray, Ireland). Glucose oxidase kit (Glucose liquicolor) and Cholesterol test kits (Cholesterol liquicolor) were purchased from Human (Human diagnostics, Wiesbaden, Germany). The total bile acid kit was purchased from BIOBASE (Jinan, Shandong, China).

### 2.2. Flour Preparation

RD43 and Hom Mali rice was grounded by using Pin Mill (Phoenix Equipment Corporation, USA) with a voltage of 50 Hz and a speed of 4800 rpm. Then the ground rice was passed through 150 µm (mesh) for removing large particles. The flour was stored in sealed plastic bags at room temperature until further analysis. To determine moisture content, each flour (5 g) was heated at 105 °C for 10 min. Moisture content was measured using the infrared moisture determination balances (KETT FD-610, Japan). The moisture content of the RD43 and Hom Mali rice flours were 11.5% and 11.0%, respectively. Total dietary fiber was carried out using the Association of Official Analytical Chemists (AOAC) method [13]. The total dietary fiber of RD43 and Hom Mali rice flours were 1.1% and 1.07%, respectively.

### 2.3. Scanning Electron Microscopy

The granular morphology of rice flour was observed using a scanning electron microscope (SEM) (JEOL, JSM-6400, Tokyo, Japan). The sample was mounted on an aluminum specimen holder using double-sided scotch tape and coated with gold at an accelerating voltage of 10 kV. The microstructure of the flour particles was scanned and photographed at various magnifications (100× and 1500×).

### 2.4. Particle Size Distributions Analysis

The particle size distributions (PSDs) of flour were analyzed using a laser particle size analyzer (Mastersizer 3000, Malvern Instruments Ltd., Worcestershire, England) with dry dispersion. The PSD was calculated using the Mastersizer 3000 software and reported as volume mean diameter (*D_4,3_*), average particle size (*D_50_*), and equivalent diameters at 10% and 90% cumulative volume (*D_10_* and *D_90_*, respectively). Three replicates were performed for each analysis.

### 2.5. Color

The color profile of flour was determined using a colorimeter (Color flex, Hunter Associates laboratory, Inc., VA, United States). Before measurements, a standard black glass and white tile were used for instrument (45°/0° geometry, 10° observer) calibration. The L*, a*, and b* parameters represent lightness, redness, and yellowness of flour, respectively. The measurement was performed in triplicate.

### 2.6. Determination of Amylose Content

The amylose content in rice flour was determined according to the previous study [4]. In brief, 0.1 g of rice flour was mixed with 1 mL of 95% ethanol and 9 mL of 1 N NaOH, and allowed to stand at room temperature for 10 min. The mixture was heated at 100 °C in a water bath for 10 min and cooled at room temperature for 2 h. The mixture was made up to 100 mL with distilled water (DW) and vigorously vortexed. A separating mixture (5 mL) was incubated with 2 mL of 1 N acetic acid, 50 mL of DW, and 2 mL of iodine solution (2.0 g potassium iodide and 0.2 g iodine in 100 mL of aqueous solution) before adjusting the final volume to 100 mL. The absorbance was recorded at 620 nm after standing for 20 min at room temperature. A calibration curve was derived using a set of pure amylose from potato.

### 2.7. X-ray Diffraction Analysis

X-ray diffraction patterns of rice flour were performed with an X-Ray Diffractometer (Bruker AXS Model D8 Discover, Leipzig, Germany) equipped with a copper tube operating at 40 kV and 45 mA, and the spectra scanned over a diffraction angle (2θ) range of 5–40° at a rate of 0.02° 2θ/second. The percentage of crystallinity was calculated using this equation [14]:

% Relative crystallinity = (Area above the smooth curve/Total diffraction area above the baseline) × 100

### 2.8. Fourier Transforms Infrared (FTIR) Spectroscopy

The FTIR of the rice flour was determined according to a previous study [10]. The rice flour samples (0.1 g, dry basis) were mixed with anhydrous KBr (0.1 g) and pressed for 20 min to obtain a transparent pellet. The pallet was transferred into the instrument and scanned in the absorption area of 450 to 4000 cm-1 by using an FTIR spectrometer (Spectrum one, PerkinElmer Life and Analytical Sciences, Shelton, CT, United States).

### 2.9. Water Absorption Index (WAI), Swelling Power (SP), and Water Solubility Index (WSI)

The hydration properties of rice flour was determined following the previous method [15]. The initial sample (*Wi*) (0.05 g) was added into 1 mL of DW, then the mixture was heated at 90 °C for 10 min in the heat block. After that, the sample was cooled in an ice water bath for 10 min and centrifuged at 3000× *g* and 4 °C for 10 min. The supernatant was separated and evaporated at 105 °C until it reached constant weight (*Ws*). The residue (*Wr*) was weighed. The results were calculated according the equation below:

WAI (g/g sample) = *Wr*/*Wi*

SP (g/g sample) = *Wr*/(*Wi* − *Ws*)

WSI (g/100g sample) = (*Ws*/*Wi*) × 100

### 2.10. Thermal Properties

The thermal properties of rice flour were determined using differential scanning calorimeter (DSC) (Netzsch DSC 204F1 Phoenix, Germany). An empty aluminum pan was used as the reference. The rice flour sample (3 mg, dry basis) was mixed with 10 μL of deionized distilled water and put into the sample pan. Then, the pan was sealed and allowed to stand at room temperature for 1 h before heating from 25 °C to 100 °C, with a heating rate of 10 °C/min. The DSC parameters, including onset temperature (*To*), peak temperature (*Tp*), conclusion temperature (*Tc*), and gelatinization enthalpy (Δ*H*), were recorded.

### 2.11. Pasting Properties

The pasting properties of rice flour samples were determined using a Rapid Visco Analyser (RVA 4500 Newport Scientific, MN, USA). In brief, the sample (3 g) was suspended in 25 g DW. Then, the suspension was continuously heated from room temperature to 95 °C at a rate of 12 °C/min. After that, the sample was held at 95 °C for 2–3 min, and finally cooled to 50 °C at a rate of 12 °C/min. Pasting temperature, peak time, peak viscosity, trough, breakdown, final viscosity, and setback were obtained from the pasting curves.

### 2.12. In Vitro Starch Digestion of Flour and Steamed Muffin

Starch digestibility of rice flour were performed according to a previous method, with minor modifications [7]. In brief, 0.5 g of rice flour or steamed muffin was boiled with 6 mL of 0.2 sodium acetate buffer, pH 6, for 15 min. Then, the cooked rice flour was equilibrated at 37 °C for 15 min in a shaker water bath (100 rpm/min). In the oral phase, the sample was incubated with 1 mL of α-amylase (250 U/mL in 0.2 M carbonate buffer, pH 7) for 15–20 s. After that, the sample was mixed with 5 mL of porcine pepsin solution (3200 U/mL in 0.02 N HCl, pH 2) and incubated for 1 h. Thereafter, gastric digesta was neutralized with 5 mL of 0.02 N NaOH and 25 mL of 0.2 M sodium acetate buffer, pH 6. Then, 5 mL of pancreatin (2 mg/mL) and amyloglucosidase (28 U/mL in 0.2 M sodium acetate buffer, pH 6) was added to the digesta at 37 °C in a shaking water bath. The sample was aliquoted at 0, 20, 30, 60, 90,120, and 180 min, and immediately heated at 105 °C for 10 min. After being centrifuged at 11,000 rpm for 15 min, the glucose release was determined using the glucose oxidase kit. The results were expressed as mg glucose/100 g sample. The incremental area under the curve (iAUC) was calculated using the trapezoidal rule. The percentage of starch fraction in the sample was reported as rapidly digestible starch (RDS; 0–20 min digestion), slowly digestible starch (SDS; 20–120 min digestion,) and undigestible starch (120–180 min digestion) [16]. Total starch was determined using a previous method [17], and factor conversion from glucose to starch was 0.9.

### 2.13. Bile Acid Binding

The bile acid binding assay was determined according to a previous method [18]. The bile acids used in this experiment were glycodeoxycholic acid, taurodeoxycholic acid, and taurocholic acid. The rice flour (1 mg/mL) was incubated with each bile acid (2 mM) in 0.1 M phosphate-buffered saline (PBS), pH 7, at 37 °C for 90 min. The mixtures were filtered through a 0.2 mm filter for separating the bound from the free bile acid. The bile acid concentration was analyzed using a bile acid analysis kit. The absorbance was measured at 540 nm. The results were reported as the percentage bile acid binding.

### 2.14. Cholesterol Micellization

The inhibition of cholesterol micellization formation was performed according to a previously established method [18]. The mixture (2 mM cholesterol, 2.4 mM phosphatidylcholine, and 1 mM oleic acid) was dissolved in methanol and dried under nitrogen before the addition of 15 mM PBS containing 6.6 mM taurocholate salt, pH 7.4. The emulsion was sonicated for 45 min using a sonicator and incubated overnight at 37 °C. The rice flour (10 mg/mL) and equivalent PBS used as a control were added to the mixed micelle solution and incubated at 37 °C for 2 h. The mixture was centrifuged at 16,000 rpm for 20 min. The cholesterol in the mixture was determined using the total cholesterol test kit. The results were calculated as percentage inhibition.

### 2.15. Steamed Muffin Preparation

Rice flour (100 g) was mixed with refined sugar (70 g), yeast (1 g), and water (160 mL) for 5 min and left at room temperature for 90 min. Finally, baking powder (3 g) was added into the mixture for 2 min. Thereafter, the muffin batter was poured into molds, which were then steamed over boiling water for 15 min. Then, the muffins were cooled at the room temperature and removed from the molds.

### 2.16. Sensory Evaluation of Steamed Muffin

The sensory evaluation of the steamed muffins was performed using untrained panelists to evaluate the acceptability for the two steamed muffins [19]. The 30 panelists, who had ever consumed steamed muffin, were recruited around Chulalongkorn University (23 females and 7 males, average age 39 ± 2 and 49 ± 4 years old, respectively). The steamed muffin was freshly prepared on the testing day. After cooling 10 min, all samples were served in a plastic box that presented three-digit numbers for identification. On the test day, the panelists were seated in individual sensory booths and asked to rinse the oral cavity with water before and between testing of samples. The analysis was performed in single replication (each subject evaluated each sample twice) as recommend by a previous report [19]. The sensory attributes were appearance, color, odor, taste, texture, hardness, and overall acceptance by using nine-point hedonic scale (1 = dislike extremely, 5 = neither like nor dislike, 9 = like extremely).

### 2.17. Statistical Analysis

The results are reported as mean ± standard error of the mean. The mean comparison was carried out by independent *t*-test. The significance of difference was considered at *p* < 0.01. The analysis was performed by using an SPSS package (SPSS 21.0 for window, SPSS Inc, Chicago, IL, USA).

## 3. Results and Discussion

### 3.1. Morphology Observation

**Figure 1:**
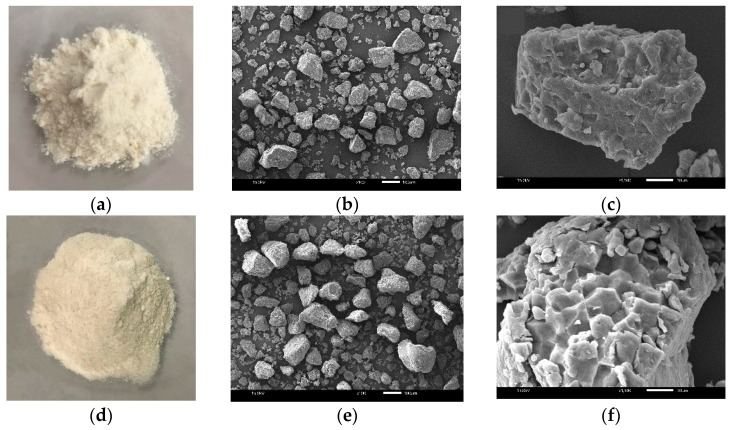
Photographs of (**a**) Hom Mali rice flour, and (**d**) RD43 rice flour. Scanning electron micrographs of Hom Mali flour, magnified (**b**) 100× and (**c**) 1500×, as well as RD43 rice flour, magnified (**e**) 100×, and (**f**) 1500×.

The photographs and scanning electron micrographs of RD43 and Hom Mali rice flour are demonstrated in Figure 1. The irregular and polyhedral shape were observed in both RD43 and Hom Mali rice flour. It has been shown that rice starches in tropical climates usually present polyhedral, round, or irregular shapes [20].

In addition, the photographs presented the aggregation of starch granules into particles with a smooth cutting surface. The obtained results of this study are consistent with the previous study following the same method of flour preparation [4]. This milling process could produce a large aggregation of flour in the presence of protein and other substances on the surface of starch granules [21].

### 3.2. Particle Size Distribution

**Figure 2:**
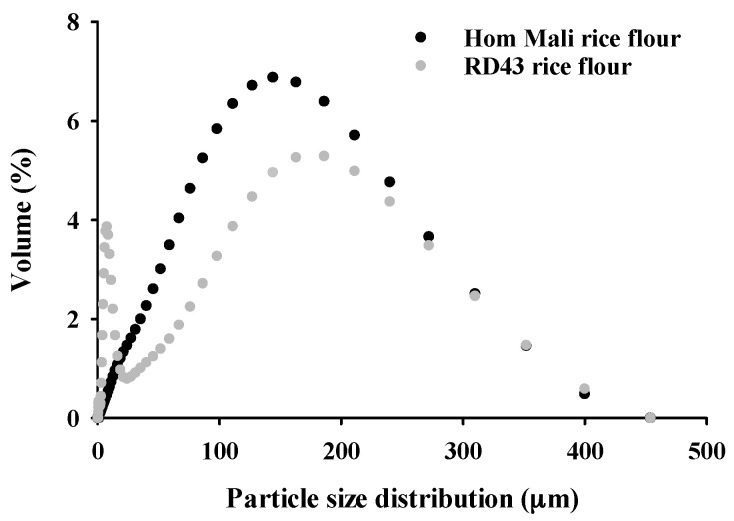
The particle size distribution by volume of rice flour.

The curve of particle size distribution of rice flour is presented in Figure 2. Hom Mali rice flour represented one peak ranging from 12.7–352.0 mm, while two peaks ranging from 4.03–9.86 µm and 11.2–400 µm were detected for RD43 rice flour. This result is consistent with a previous study showing two different particle diameters of riceberry rice obtained from the same milling equipment [4]. The first peak may be due to the damaged starch granule remnant [21], whereas the second peak demonstrates the aggregation as indicated by SEM images.

As shown in Table 1, the value of *D_4,3_*, *D_10_*, and *D_50_* of RD43 rice flour was significantly lower than that of Hom Mali rice flour, suggesting that RD43 rice flour had smaller average particle size. The finer particle size of flour resulted from less grain strength [22]. The previous study suggests that the small particle size of flour might produce fine and massive textures of finished products [5].

### 3.3. Color of Rice Flour

As shown in Table 1, RD43 rice flour had significantly lower lightness (L*) and higher redness (a*) and yellowness (b*) when compared to Hom Mali rice flour. The rice flour with a greater degree of yellowness and less intensity in lightness might result from different rice cultivars, storage time, and the exposure to high temperatures during the milling process [5,23]. In this study, the rice flour was prepared and kept in the same condition. This suggests that the difference in color values is most likely attributed to the rice cultivar.

### 3.4. The Amylose Content of Rice Flour

As demonstrated in Table 1, the amylose content of Hom Mali and RD43 rice flour was 16.38% ± 0.65% and 18.62% ± 0.27%, respectively, suggesting that they were classified as low-amylose (12–20%) according to categorization of rice based on the amylose content [24]. Nevertheless, the RD43 rice flour presented higher amylose content than the Hom Mali rice flour. The variation of amylose content depends on cultivars and botanical source [5]. The different content of amylose directly affects gelatinization, pasting behavior, swelling power, crystallinity, and starch digestibility in relation to the content of resistant starch (RS) [6,25]. In food manufacturing, rice flour containing low amylose promotes an increase in moistness, chewiness, and soft texture [8], whereas rice flour containing high amylose rice contributes to increase firmness and crispness in food products [26].

### 3.5. X-ray Diffraction

**Figure 3:**
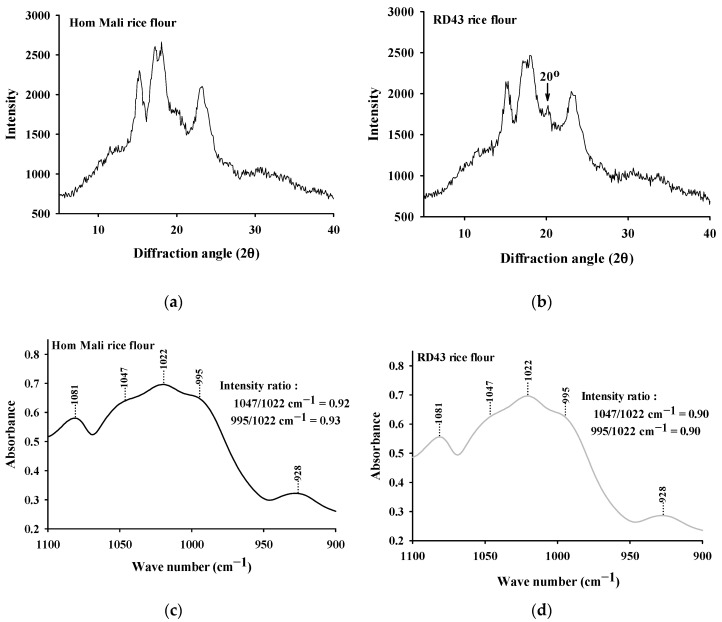
X-ray diffraction pattern of (**a**) Hom Mali and (**b**) RD43 rice flour. Fourier transform infrared (FTIR) spectra of (**c**) Hom Mali and (**d**) RD43 rice flour.

As presented in Figure 3a,b, RD43 and Hom Mali rice flour presented diffraction peaks at 2θs of 15° and 23°, while they exhibited unresolved peaks at 17° and 18°. This peak pattern is the A-type crystalline structure known as cereal starch, such as wheat, maize, and rice. The A-type crystalline structure containing densely packed forms of double helices in a monoclinic unit cell is more stable conformationally than other crystalline structures [5]. Interestingly, the apparent diffraction peak at 20° was only observed in RD43 rice flour (Figure 3a). This peak indicates the V-type crystalline structure in relation to the existence of amylose–lipid complexes in the rice flour [27].

According to the calculation of the X-ray diffraction pattern, the degree of crystallinity in RD43 rice flour (24.55%) was significantly lower than observed in Hom Mali rice flour (26.8%). This result suggests that the lower degree of relative crystallinity in RD43 rice flour results from the higher presence of amorphous area [28].

### 3.6. FTIR Spectroscopy

The FTIR spectra of Hom Mali rice and RD43 rice flour are illustrated in Figure 3c,d. The absorption bands in the region 1100–900 cm^−1^ represent the sensitive change in the starch structure. The observed peaks at 1081 and 1022 cm^−1^ indicate stretching of the anhydroglucose C–O ring, which related to polysaccharide molecules [29]. In addition, the peak at 928 cm^−1^ was assigned to polysaccharides with α-1,4 glycosidic linkage [30]. Several studies have reported that observed absorption bands at 1047 and 995 cm^−1^ are the crystalline region of starch, whereas a band at 1022 cm^−1^ is characterized as an amorphous region of starch [6,31]. In particular, the intensity ratio of 1047/1022 and 995/1022 cm^-1^ could refer the association between amount crystallinity and amorphous structure [31,32]. Comparing with Hom Mali rice flour, RD43 rice flour expressed the lower intensity ratio of 995/1022 and 1047/1022 cm^−1^, indicating the lower degree of double helices and ordered structure [32]. The lower intensity ratio of 1047/1022 cm^−1^ especially is interpreted as a high amount of amylose with a low amylopectin content [6]. This result suggests that RD43 rice flour has lower degree of crystallinity than Hom Mali rice flour.

### 3.7. Hydration Properties of Rice Flour

The WSI represents the ability of components to dissolve with water under an excess water condition. As demonstrated in Table 1, the lower WSI was obtained from RD43 rice flour, as compared to Hom Mali rice flour. The low WSI of RD43 rice flour may facilitate a firm and dense internal structure of food products [33]. The WAI and SP represent the interactions between the water molecules and the starch chains in the crystalline and amorphous regions. In this study, RD43 rice flour had a lower WAI and SP than Hom Mali rice flour (Table 1). Previously, the significant difference in WAI and SP was detected in various rice cultivars [4,5]. In addition, the WAI and SP demonstrated a negative correlation with the amylose content [6,34]. The lower SP and WAI of RD43 rice flour may be attributable to the higher amylose content.

### 3.8. Thermal Properties of Rice Flour

The differential scanning calorimetry (DSC) curves and values are shown in Supplementary Figure S1. As demonstrated in Table 1, RD43 rice flour had a significantly lower peak temperature (*T_p_*) and conclusion temperature (*T_c_*) than Hom Mali rice flour. There was no significant difference in onset gelatinization temperature (*T_o_*) and gelatinization enthalpy (Δ*H*) between RD43 rice flour and Hom Mali rice flour. The thermal properties are normally affected by the chemical composition, the origin of starch, moisture, the presence of other biomaterials, and processing and pretreatment conditions [35]. Previous studies have revealed a positive correlation between *T_p_*, *T_c_*, and crystallinity in rice flour [5]. The greater degree of crystallinity causes an increase in transition temperature due to more resistance and the stability of the granule structure for gelatinization [36]. Therefore, the lower transition temperature values of RD43 rice flour may be related to the presence of a lower degree of crystallinity.

### 3.9. Pasting Properties of Rice Flour

**Figure 4:**
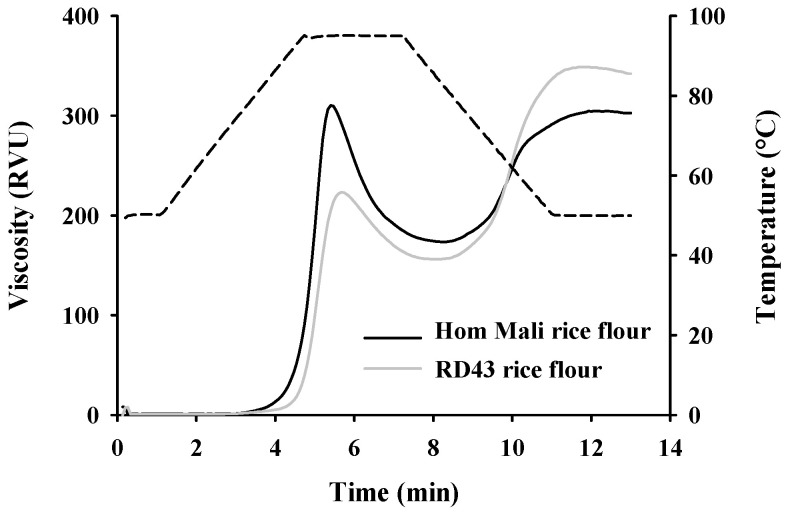
Pasting properties pattern of Hom Mali and RD43 rice flour.

The pasting curves of RD43 rice and Hom Mali rice flour are presented in Figure 4. When compared to Hom Mali rice flour, RD43 rice flour showed a lower value for peak, trough, and breakdown viscosity, whereas it exhibited higher value of peak time, pasting temperature, setback, and final viscosity (Table 1). These results suggest that RD43 rice flour had lower capacity to form a gel (low peak viscosity), which arises from a greater resistance to heating and shear stress (low breakdown) leading to highly stable form during cooking. RD43 rice flour also requires a higher temperature and longer time for the pasting process (high pasting temperature and peak time) than Hom Mali rice flour. In the cooling stage, RD43 rice flour had higher stability for the cooked paste (high final viscosity) and tendency toward retrogradation (high setback) than Hom Mali rice flour. The exhibition of larger final viscosity and setback value may be responsible for a huge tendency for retrogradation in food products during storage [4], whereas the high-setback rice flour provides more food structure. This rice flour property is appropriately recommended for fried snacks, stick rice noodles, and pasta noodles [37]. The results from pasting properties indicate that RD43 rice flour may be more enhance the structure of food products than Hom Mali rice flour.

The distinct pasting behavior of rice flour is influenced by cultivars, resulting in variation of the undigestible starch and amylose content [5,38]. Higher undigested starch content in rice flour may involve a compact linear structure that responsible for high setback and final viscosity [39]. Moreover, the presence of amylose leads to decrease negative charges of the phosphate group in an amylopectin structure and binding of the hydrogen bond in water, which inhibits the swelling of starch granules [26]. Ye et al. described that rice flour containing high amylose content contributes to increased pasting temperature, setback, and final viscosity, due to the flour’s lower swelling ability and molecule rearrangement [38]. Also, the amylose–lipid complex may influence pasting properties by increasing the hydrophobicity of starch granules, resulting in an increase in granule swelling and amylose leaching [36]. Consequently, this interaction causes a reduction of peak and breakdown viscosity and an increase in setback and final viscosity [36,37]. In addition, storage time, temperature during milling process, and environment are other factors on different pasting properties of the rice flour [40,41,42].

### 3.10. In Vitro Starch Digestion

**Figure 5:**
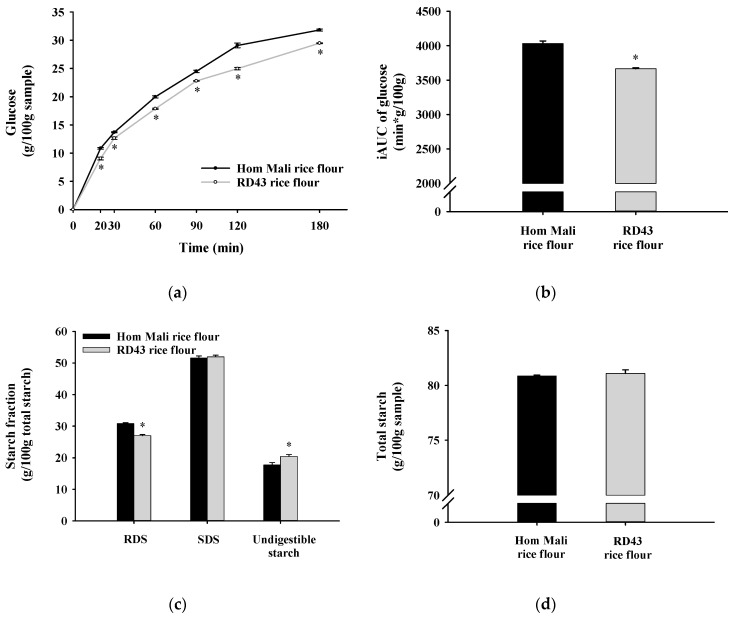
The glucose release during in vitro starch digestion from (**a**) rice flour, (**b**) incremental area under the curve (iAUC) of glucose, (**c**) starch fraction, and (**d**) total starch of Hom Mali and RD43 rice flours. Data are expressed as mean ± standard error of the mean, *n* = 3. * *p* < 0.01 compared to Hom Mali rice flour. RDS: rapidly digestible starch, SDS: slowly digestible starch.

The starch digestibility of RD43 and Hom Mali rice flour is demonstrated in Figure 5a. The release of glucose was observed from all samples over 20 min, and it gradually increased after 30 min of digestion. The results showed that RD43 rice flour exhibited lower glucose release and iAUC than Hom Mali rice flour throughout 180 min (Figure 5b). Moreover, RD43 rice flour presented a significantly lower percentage of RDS and higher percentage of undigestible starch than Hom Mali rice flour (Figure 5c). As shown in Figure 5d, both rice flours had a similar percentage of SDS, total starch (Table 1), and dietary fiber. It has been shown that the consumption of diets containing a low percentage of RDS and the high content of undigestible starch causes a reduction of postprandial blood glucose [43]. The present findings can be explained by the presence of amylose and amylose–lipid complex content in RD43 rice flour [27]. A rigid structure of amylose causes less accessibility to enzymatic action, such as α-amylase and amyloglucosidase [25], which provides a significant positive impact towards controlling postprandial blood glucose [44].

### 3.11. Bile Binding Acid and Cholesterol Micellization

The percentage of bile acid binding and the inhibition of cholesterol micellization of RD43 rice and Hom Mali rice flour are demonstrated in Table 2. The RD43 rice flour had greater ability to bind bile acids (taurocholic acid, taurodeoxycholic acid, and glycodeoxycholic acid). In addition, RD43 rice flour had greater ability to disrupt the formation of cholesterol micelles than Hom Mali rice flour. Due to the similar dietary fiber content, the inhibitory action of RD43 rice flour may be attributed to the existence of greater amylose content. Previous studies have found that the helical structure of amylose could form complexes with bile salts and cholesterol [45,46]. It has been shown that the bile acid binding property and the disruption of cholesterol micellization is an attractive target for reducing cholesterol absorption into the small intestine [18]. It suggests that RD43 rice flour may help reduce the amount of cholesterol in blood circulation. Further research is warranted to resolve this issue in human studies.

### 3.12. Starch Digestibility and Sensory Evaluation of Steamed Muffins

**Figure 6:**
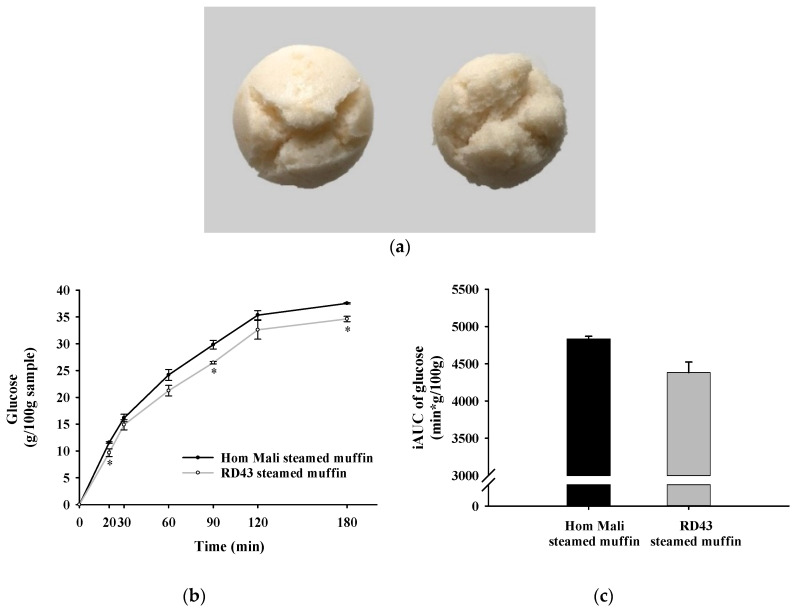
Photographs of (**a**) rice flour steamed muffin (left: Hom Mali steamed muffin, right: RD43 steamed muffin). (**b**) The glucose release during in vitro starch digestion from rice flour muffins, and (**c**) incremental area under the curve (iAUC) of glucose. Data are expressed as mean ± standard error of the mean, *n* = 3. * *p* < 0.01 compared to Hom Mali steamed muffin.

**Figure 7:**
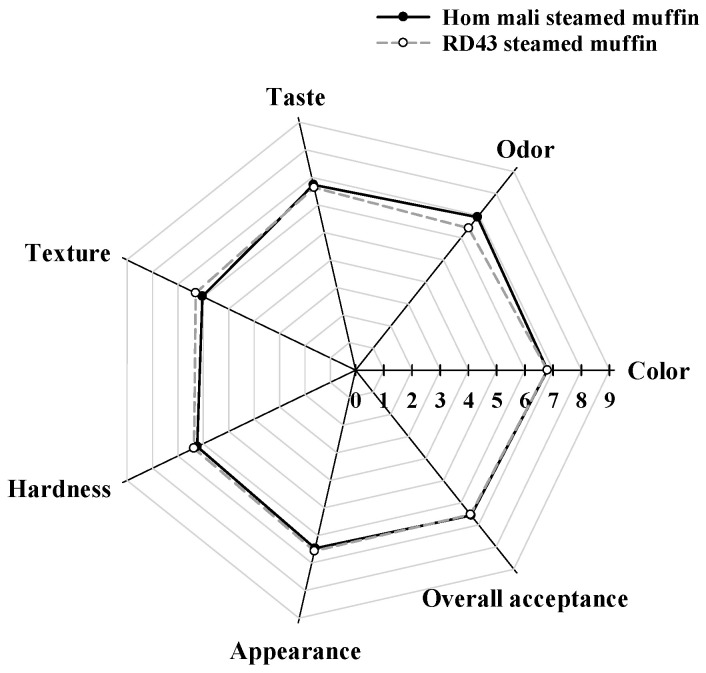
Sensory analysis of Hom Mali and RD43 rice steamed muffin. Data are expressed as the mean.

The steamed muffins of Hom Mali and RD43 rice flour are presented in Figure 6a. Compared to the Hom Mali steamed muffin, the RD43 steamed muffin had significantly lower glucose release at 20, 90, and 120 min (Figure 6b). In addition, the iAUC of the RD43 steamed muffin was significantly lower than that of Hom Mali steamed muffin (Figure 6c). This indicates that the RD43 steamed muffin shows a greater ability to slow down starch digestibility than the Hom Mali steamed muffin. Our findings are consistent with previous studies that bread made from riceberry rice flour had lower glucose release than Hom Mali bread [4]. We suggest that RD43 flour may be a promising ingredient for reducing the glycemic index (GI) of food products, which helps to control postprandial blood glucose [43]. The sensory evaluation of steamed muffin made from Hom Mali and RD43 rice is shown in Figure 7. Sensory parameters of the steamed muffins included appearance, color, odor, texture, hardness, taste, and overall acceptability. The findings showed that sensory scores of the steamed muffins made from RD43 and Hom Mali rice showed no significant difference in any evaluated attributes.

## 4. Conclusions

RD43 rice flour presented a higher amylose content with a smaller particle size than Hom Mali rice flour. The results from X-ray diffraction indicated the presence of amylose–lipid complexes in RD43 rice flour. This structure influences hydration, thermal properties, and pasting properties of RD43 rice flour. Furthermore, RD43 rice flour had a greater ability to decrease starch digestibility, bind bile acid, and disrupt the formation of cholesterol micellization than Hom Mali rice flour. Steamed muffin made from RD43 flour had lower starch digestibility without affecting the sensory acceptability. Based on our findings, RD43 rice flour may be used as an alternative ingredient for lowering the glycemic index of food products.

## Figures and Tables

**Table 1:** The particle size distribution, color profile, and amylose content, as well as hydration, thermal and pasting properties of Hom Mali and RD43 rice flour.

Physicochemical Properties	Rice Flour	
	Hom Mali	RD43
Particle size distribution (µm)		
Volume mean diameter (*D_4,3_*)	130.00 ± 0.58	103.00 ± 0.15 *
Equivalent diameters at 10% cumulative volume (*D_10_*)	23.33 ± 0.13	5.41 ± 0.33 *
Average particle size (*D_50_*)	115.67 ± 0.33	73.07 ± 0.17 *
Equivalent diameters at 90% cumulative volume (*D_90_*)	259.67 ± 1.73	257.00 ± 2.52
Color		
Lightness (L*)	91.09 ± 0.06	89.4 ± 0.02 *
Redness (a*)	0.01 ± 0.02	0.16 ± 0.02 *
Yellowness (b*)	5.38 ± 0.07	6.29 ± 0.07 *
Amylose content (%) ^a^	16.05 ± 0.39	19.04 ± 0.30 *
Hydration properties ^a^		
WSI (g/g sample)	9.33 ± 0.13	7.33 ± 0.13 *
WAI (g/g sample)	13.06 ± 0.13	12.41 ± 0.07 *
SP (g/100g sample)	13.68 ± 0.11	12.35 ± 0.16 *
Thermal properties		
*T_o_* (°C)	65.63 ± 0.07	65.07 ± 0.26
*T_p_* (°C)	71.20 ± 0.15	70.23 ± 0.22 *
*T_c_* (°C)	76.6 ± 0.10	75.20 ± 0.31 *
Gelatinization enthalpy (J/g)	7.85 ± 0.28	6.89 ± 0.31
Pasting properties		
Peak viscosity (RVU)	310.38 ± 3.71	223.50 ± 0.35 *
Trough (RVU)	173.33 ± 0.94	156.17 ± 0.94 *
Breakdown (RVU)	137.04 ± 2.77	67.33 ± 1.30 *
Final viscosity (RVU)	302.71 ± 2.77	342.08 ± 3.30 *
Setback (RVU)	129.38 ± 1.38	185.92 ± 2.36 *
Peak time (min)	5.43 ± 0.05	5.70 ± 0.05 *
Pasting temperature (°C)	84.05 ± 0.07	89.73 ± 0.04 *
Total starch (%)	81.43 ± 0.58	80.72 ± 0.34

Values are expressed as mean ± standard error of the mean, *n* = 3. * *p* < 0.01 compared to Hom Mali rice flour. ^a^ Data are based on dry basis. WSI: water insolubility index, WAI: water absorption index, SP: swelling power, *T_o_*: onset temperature, *T_p_*: peak temperature, *T_c_*: conclusion temperature, Δ*H*: enthalpy gelatinization. RVU: Rapid Visco Unit.

**Table 2:** Effects of Hom Mali and RD43 rice flour on bile acid binding and the inhibition of cholesterol micellization.

Rice Flour	% Bile Acid Binding			% Cholesterol Micellization Inhibition
	Taurocholic Acid	Taurodeoxycholic Acid	Glycodeoxycholic Acid	
Hom mali	2.51 ± 0.23	NA	NA	9.22 ± 0.48
RD43	7.45 ± 0.36 *	36.35 ± 0.19 *	11.61 ± 0.12 *	12.95 ± 0.52 *

Values are expressed as mean ± standard error of the mean, *n* = 3. * *p* < 0.01 compared to Hom Mali rice flour. NA: no activity.

## Supplementary Materials

The following are available online at https://www.mdpi.com/2304-8158/9/12/1912/s1, Figure S1: Differential scanning calorimetry (DSC) curves of (**a**) Hom Mali and (**b**) RD43 rice flour.
