# Supplementary material for: Physicochemical and Functional Characteristics of RD43 Rice Flour and Its Food Application
Source: Foods. 2020 Dec 21;9(12):1912. doi: 10.3390/foods9121912 (PMC7767328; doi:10.3390/foods9121912)
Supplement: Supplementary file 1 [file foods-09-01912-s001.pdf]

foods

Physicochemical and functional characteristics of RD43 rice flour and its food application

Phim on Suklaew, Charoonsri Chusak and Sirichai Adisakwattana *

Phytochemical and Functional Food Research Unit for Clinical Nutrition, Department of Nutrition and Dietetics, Faculty of Allied Health Sciences, Chulalongkorn University, Bangkok, 10330, Thailand;

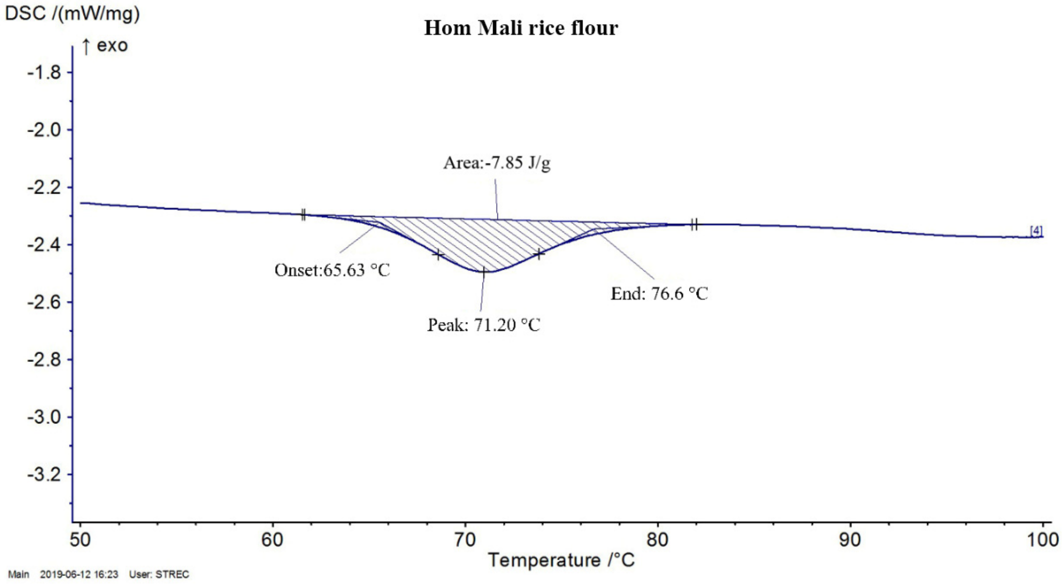

(a)

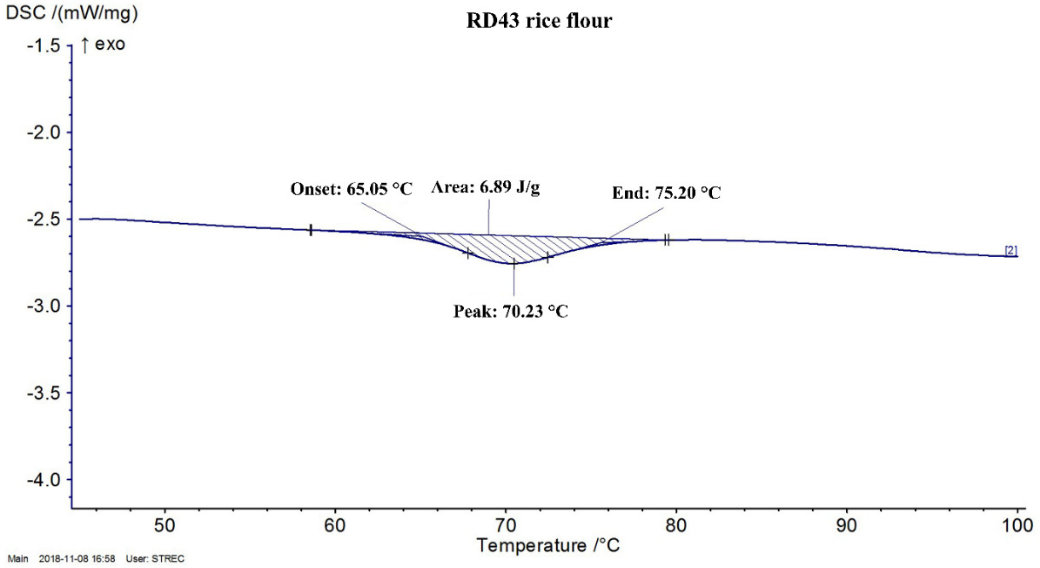

(b)

Supplementary Figure 1. Differential scanning calorimetry (DSC) curves of (**a**) Hom Mali and (**b**) RD43 rice flour.
